# Supplementary material for: Selective feeding in Southern Ocean key grazers—diet composition of krill and salps
Source: Commun Biol. 2021 Sep 10;4:1061. doi: 10.1038/s42003-021-02581-5 (PMC8433442; doi:10.1038/s42003-021-02581-5)
Supplement: Supplementary file 2 — Supplementary Information [file 42003_2021_2581_MOESM2_ESM.pdf]

Supplementary Information for
Selective feeding in Southern Ocean key grazers – diet composition of krill and salps

Nora-Charlotte Pauli, Katja Metfies, Evgeny A. Pakhomov, Stefan Neuhaus, Martin Graeve, Philipp Wenta, Clara M. Flintrop, Thomas H. Badewien, Morten H. Iversen, and Bettina Meyer

Supplementary Material and Methods

Blocking probe design

Probes to block the predator DNA for krill stomach samples were designed to bind between the applied primers using the ARB software tool[1]. In a first step, possible probes were evaluated based on the following criteria: a) a length of 18–25 bp, b) zero mismatches with Antarctic krill (*Euphausia superba*) and its closest relatives (Eucarida, following WORMS taxonomy), c) a melting temperature (Tm) corresponding to that of the primers, and d) at least 2–3 mismatches with the main target prey taxa.

This evaluation resulted in five potential probes, which were subsequently tested in temperature gradient polymerase chain reactions (PCR) using five different concentrations at a temperature range from 50 °C to 60.4 °C using DNA from the same krill individual. Samples were sequenced in triplicates along with a control not including the blocking probe. Overall, the samples including a blocking probe showed a considerably lower amount of total reads compared to the control (Supplementary Figure 8a). Probes #1 and #2 showed no difference in the amount of predator DNA compared to the control (Supplementary Figure 8b), while Probe #3 and #4 reduced the amount of predator DNA by about half. Probe number 5 showed similar results to #3 and #4, however only one replicate had enough reads to pass the quality filtering.

In a second step, one of the successfully tested probes (#4) was modified by adding a C3-spacer at both ends to prevent the probe from being digested by the DNA polymerase during amplification. In addition, a reverse complementary probe was designed based on the hypothesis, that the amount of amplified predator DNA could be further reduced by blocking both complementary strands of the predator DNA. Probes were added to a PCR mix using DNA from the same krill individual as before and sequenced in triplicates in the following set-up: a) only modified probe, b) only reverse complementary probe, c) a combination of both. This resulted in less than 100 reads for the reverse complementary probe, as well as for the combinatory approach. The modified probe with C3-spacers yielded similar results as the initial probe test runs. Based on the obtained results, for the final sequencing of the krill stomach content samples, probe #4 without C3-spacer was used.

Supplementary Results

Composition of stomach content, fecal pellets and the ambient plankton community

An exploratory principal component analysis (PCA) accounted for 44% of explained variation in the first two dimension of the refined dataset, while the remaining dimensions each explained less than 5% of the variation. A quantitative PCA using only amplicon sequence variants (ASV) with significant differences between or within sampling groups, or compositionally associated ASVs, revealed more distinctly separated clusters of both species within the stomach content and fecal pellets groups, respectively (Supplementary Figure 2). However, the explanatory power here was limited to 29.8% of explained variance in the first two dimensions (19.6% and 10.2%, respectively).

Unsupervised clustering using the Euclidean distances was performed to further evaluate the observed clusters. The two main clusters separated plankton and fecal pellets from stomach content samples, respectively (Supplementary Figure 3). Within the first main cluster, the plankton community formed one distinct group and fecal pellets another. Two salp samples clustered more closely within the krill fecal pellet samples adjacent to a krill sample taken from the same drift trap (DF 10). Moreover, three krill fecal pellet samples from drift traps DF3, and DF4 clustered with the salp pellet group. The second main cluster consisted exclusively of stomach content samples and showed a rough separation into salp and krill samples; however, there were several overlaps and multiple sub clusters. The left branch of the second main cluster separated two main groups mainly including samples from both krill and salps taken at two adjacent stations at Deception Island and in the Bransfield Strait West region (St. 24 & 25).

Mapping other metadata variables on the PCA biplot revealed no apparent clusters by station or region (Supplementary Figure 4), indicating that the separation by sampling groups based on species and samples type (stomach content, fecal pellets, plankton) adequately explained the structure of the data. Moreover, the clustering patterns were robust to data manipulation when rare taxa (<1%) were removed.

The ambient plankton community showed little differences between the single samples taken at different stations (Supplementary Figure 1). Two samples from the Bransfield Strait East and Antarctic Sound showed fewer dinoflagellates (Dinophyceae) and diatoms (Bacillariophyta) and a higher share of Pelagophyceae compared to the other plankton samples. In contrast to the stomach content of krill, salp stomach samples showed some regional patterns. One group of salp stomach samples from stations 24 and 25 was characterized by a high relative abundance of small flagellates (>60%; Filosa-Thecofilosea, Ebriida), which coincides with the clusters observed using Euclidean distances. Salp samples from the South Shetland Island (Station 35) included more than 50% of the parasitic dinoflagellate group Syndiniales. Single salp stomachs almost entirely consisted of copepods (Arthropoda).

Finally, pairwise comparisons of all five groups to test for reproducibly different taxa between groups (effect size>1, using 128 Monte Carlo replicates from a Dirichlet distribution) supported the main patterns concluded from the relative abundances. Diatoms (Bacillariophyta), dinoflagellates

(Dinophyceae), and the parasitic dinoflagellates group Syndiniales differed significantly between the ambient plankton community and the fecal pellets produced by krill and salps. Salps (Tunicata) were the only taxon that differed between krill and salp stomachs.

Fatty acid composition

Polyunsaturated fatty acids (PUFA, n-3 and n-6) contributed 37.3% to the total fatty acids in krill, and 29.9% in salp tissue. Monounsaturated fatty acids (MUFA) accounted for 21.6% in krill, and 17.6% in salp tissue, respectively. The predominant fatty acids in both species were 16:0, as well as the PUFA 20:5(n-3) and 22:6(n-3). In addition, 18:1(n-7), 18:1(n-9) and 14:0 accounted each for more than 5% of total fatty acids. Wax esters accounted for less than 1% in krill samples, while they were more abundant in salp samples (median = 3.2%), with three samples showing amounts of 10–20%, and one single sample 34.9%. Fatty alcohols mainly included 16:0 in krill, and 14:0, 16:0, 18.1(n-9) and 20:1 in salp tissue.

Supplementary Table 1: Taxa, which occurred with a relative abundance of 0.5 – 1.5% within each of the 18S sequencing libraries of the five sampling groups (plankton, krill stomach, salp stomach, krill fecal pellets, salp fecal pellets). The respective share of each taxon per group is shown as percentage.

Taxon	Plankton	Krill stomach	Salp stomach	Krill pellets	Salp pellets
Acantharea	1.37	-	-	-	0.79
Annelida	-	0.56	-	-	-
Ascomycota	-	-	-	-	1.14
Bolidophyceae	-	-	0.58	-	1.37
Cercozoa_X	-	-	-	-	0.55
Chaetognatha	-	0.67	-	-	-
Chrysophyceae	-	-	0.68	0.80	-
Filosa-Imbricatea	-	0.88	-	-	-
Filosa-Thecofilosea	1.27	-	-	-	-
Gregarinomorphea	-	-	0.55	-	-
Mamiellophyceae	-	1.24	-	-	0.58
MAST-1	-	-	-	0.54	-
MAST-3	1.17	0.93	-	-	-
Mollusca	-	1.06	-	-	-
Pelagophyceae	-	1.09	-	-	-
Phyllopharyngea	0.65	1.25	-	-	-
Picozoa_X	-	0.89	-	-	-
Prymnesiophyceae	-	-	0.55	0.52	-
Pyramimonadales	0.80	-	-	-	-
RAD-B	-	1.19	-	-	-
Spirotrichea	-	-	0.60	1.04	-

Supplementary Table 2: Fatty acids identified from the tissue of krill (*Euphausia superba*) and salps (*Salpa thompsoni*) as mean percentage of total fatty acids given with standard deviation.

**Fatty acid**	***Euphausia superba***			***Salpa thompsoni***		
14:0	8.67	±	4.96	8.01	±	6.34
14:1(n-5)	0.16	±	0.07	0.08	±	0.00
15:0	0.50	±	0.71	3.11	±	1.62
16:0	25.37	±	10.35	27.78	±	14.43
16:1(n-7)	5.69	±	3.73	4.81	±	3.57
16:1(n-5)	0.40	±	0.26	1.59	±	1.63
16:2(n-4)	0.62	±	0.35	0.04	±	0.00
16:3(n-4)	0.43	±	0.26	0.06	±	0.00
16:4(n-1)	1.37	±	0.74	0.25	±	0.33
18:0	1.29	±	0.82	6.24	±	8.27
18:1(n-9)	8.31	±	5.16	5.99	±	6.95
18:1(n-7)	6.60	±	3.79	5.11	±	3.55
18:1(n-5)	0.17	±	0.19	0.31	±	0.38
18:2(n-6)	1.62	±	1.20	1.66	±	1.88
18:3(n-3)	0.77	±	0.62	0.48	±	0.75
18:4(n-3)	3.08	±	2.92	1.91	±	2.21
20:1(n-11)	0.13	±	0.00	0.54	±	0.51
20:1(n-9)	0.73	±	0.51	1.19	±	1.00
20:1(n-7)	0.25	±	0.15	1.63	±	2.37
20:2(n-3)	0.35	±	0.40	0.03	±	0.00
20:2(n-6)	0.10	±	0.00	1.54	±	5.16
20:3(n-3)	0.17	±	0.00	0.49	±	0.58
20:4(n-3)	0.38	±	0.31	0.16	±	0.29
20:5(n-3)	20.85	±	12.08	11.62	±	8.24
22:1(n-11)	0.13	±	0.00	1.74	±	1.51
22:1(n-9)	0.60	±	0.54	0.14	±	0.42
22:1(n-7)	0.18	±	0.10	0.14	±	0.00
22:2(n-6)	0.12	±	0.13	0.39	±	0.70
22:5(n-3)	0.37	±	0.21	0.15	±	0.42
22:6(n-3)	10.45	±	5.88	12.69	±	7.56

Supplementary Table 3: All sampled regions with respective stations, latitude, longitude, and deployed sampling devices. Sampling devices included drifting sediment traps (DF), a conductivity, temperature and depth water sampler (CTD), Isaacs-Kidd Midwater Trawls (IKMT) and Rectangular Midwater Trawls (RMT). Stations where additional plankton samples were collected for a vertical profile at multiple depths are highlighted with an asterisk.

Region	Station	Latitude	Longitude	Date	Sampling device	Sampling depth [m]
**Antarctic Sound**	90	-63.77527	-57.09111	16.04.2018	DF	100/300
	98	-63.85639	-56.67639	17.04.2018	DF	100/300
	103	-62.75160	-56.50012	19.04.2018	CTD, IKMT	171
**Elephant Island**	50	-61.03126	-55.00098	07.04.2018	IKMT	48
	51	-61.06370	-54.75805	08.04.2018	IKMT	35
	54	-61.04694	-54.98611	08.04.2018	DF	100/300
	55*	-60.99710	-54.83685	08.04.2018	CTD, RMT	3/50/100/200
	56	-60.74813	-55.50912	09.04.2018	CTD	30
	111	-60.93648	-55.04189	24.04.2018	IKMT	20
	118	-61.01556	-55.06722	25.04.2018	DF	100/300
	119	-61.19583	-54.85111	26.04.2018	DF	100/300
	120	-61.16083	-54.85028	27.04.2018	DF	100/300
	121	-60.99833	-54.89167	28.04.2018	DF	100/300
	122	-61.06278	-55.01639	30.04.2018	DF	100/200/300
**Bransfield Strait East**	105	-62.50161	-53.99577	19.04.2018	CTD	20
	106*	-62.01472	-54.05694	20.04.2018	CTD, IKMT, DF	14/25/100/172/200
**Bransfield Strait West**	18	-62.53224	-59.41006	26.03.2018	IKMT, CTD	180
	19	-62.74975	-59.98112	26.03.2018	CTD	20
	23	-62.58368	-59.70203	27.03.2018	IKMT	170
	24	-62.56178	-59.92788	28.03.2018	IKMT	50
**South Shetland Islands**	35	-61.50421	-60.52402	04.04.2018	CTD, IKMT	100
	36	-61.74034	-59.98894	04.04.2018	CTD, IKMT	170
	38	-61.49985	-59.49887	05.04.2018	CTD	30
	41*	-61.74239	-59.01837	06.04.2018	CTD, IKMT	3/60/100/170/200
	42	-61.75152	-57.49525	06.04.2018	CTD	15
**Deception Island**	25*	-62.97389	-60.47721	29.03.2019	CTD	3/50/56/100/200
		-62.96615	-60.46593	30.03.2018	IKMT	40

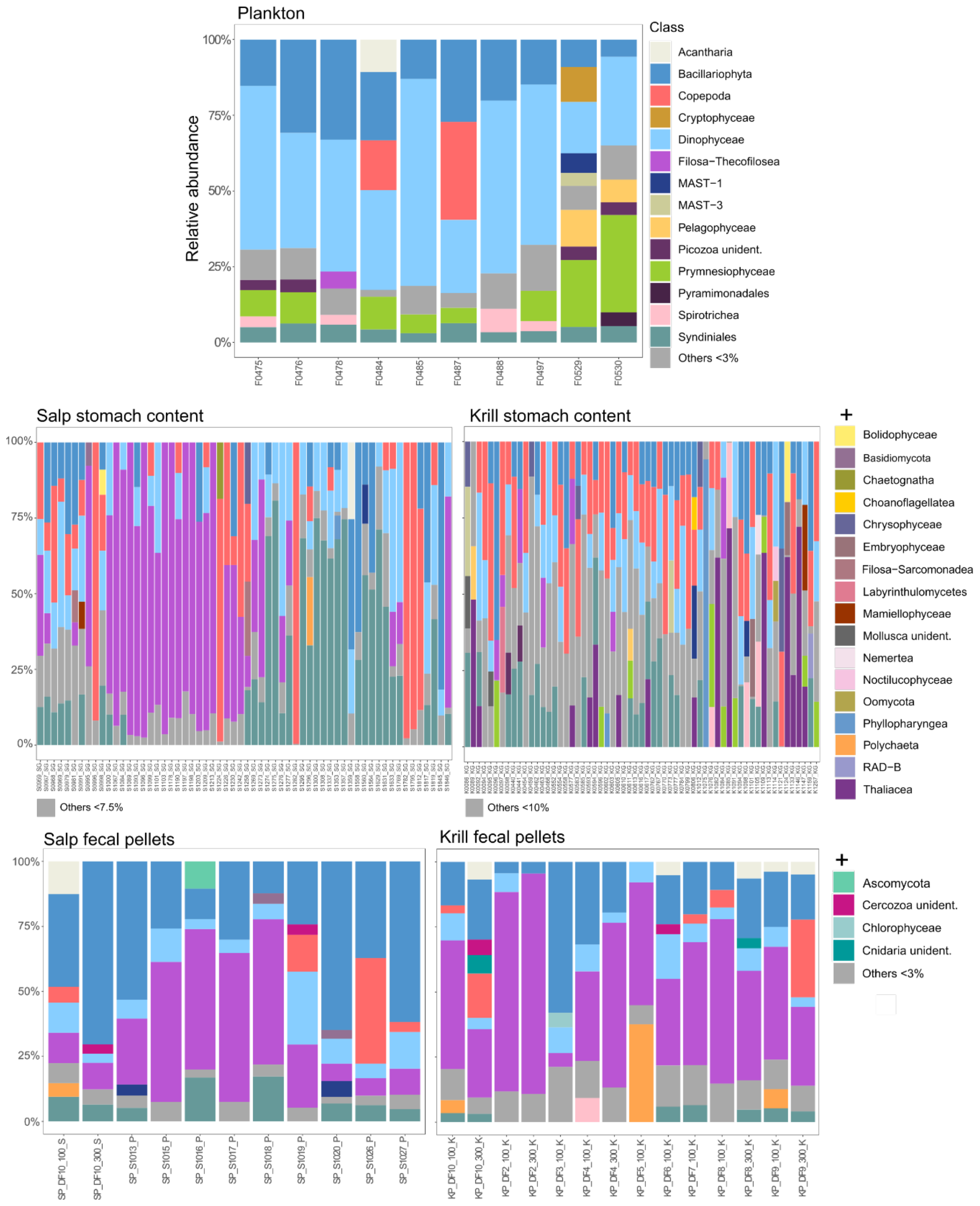

Supplementary Figure 1: Composition of the 18S sequencing libraries per sample in the five sampling groups. Relative abundance of taxa aggregated on the level of ‘Class’ from the filtered dataset containing 156 samples and 1765 ASVs were used. Bars represent single samples as depicted on the x-axis, the relative abundance of each class is shown in percent on the y-axis. Rare taxa are group into ‘Others’, representing taxa with a relative abundance of less than 3% for the plankton community, salp and krill fecal pellets, 7.5% for salp stomach content samples and 10% for krill stomach content samples, respectively.

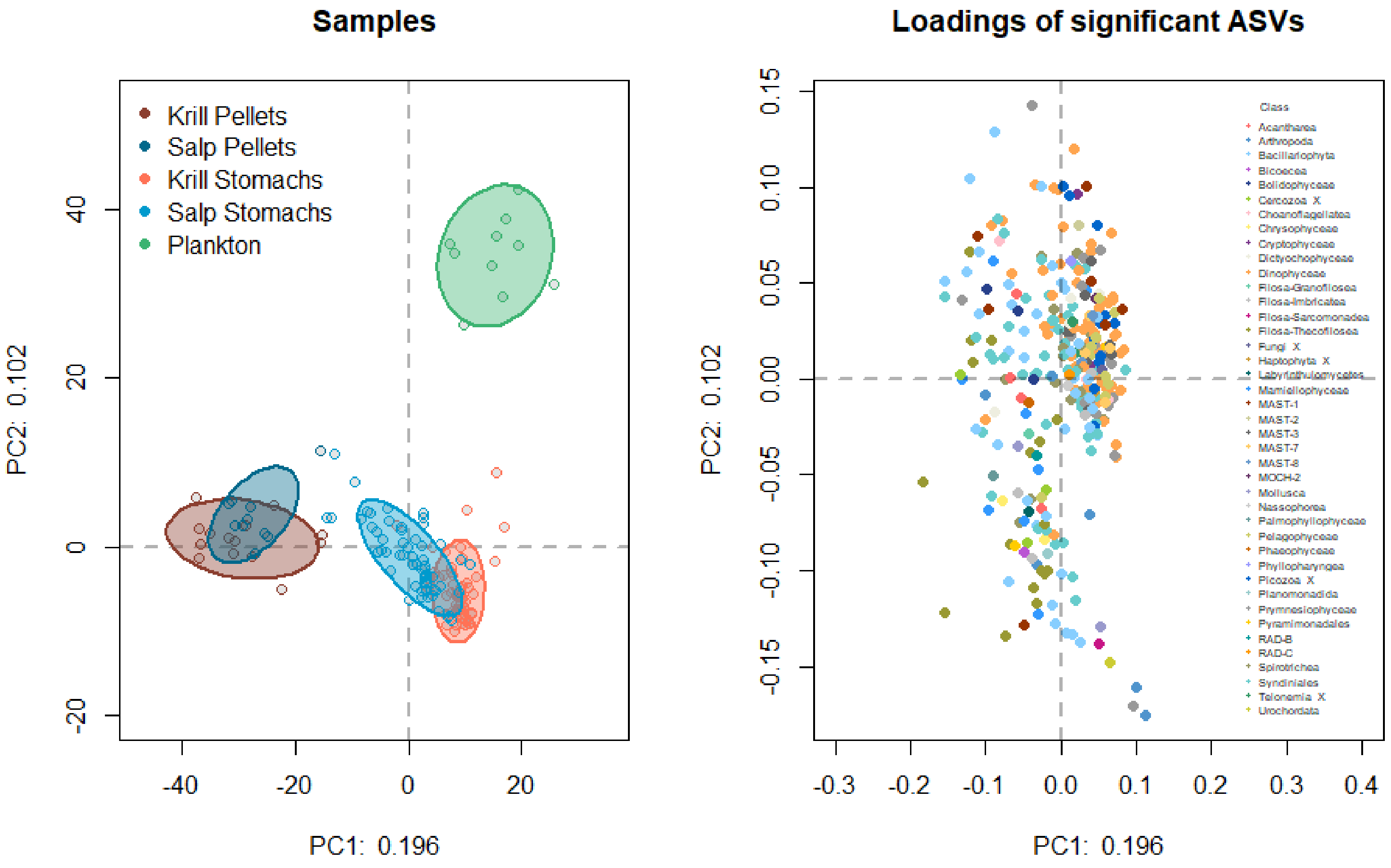

Supplementary Figure 2: Quantitative principal component analysis of the 18S sequencing libraries using a subset of the data including ASVs with an effect size >1 within or between any of the groups or with a correlation metric ρ >0.5. The left plot shows the samples of the subset with the sampling groups highlighted by ellipses of a 75% confidence interval: Krill fecal pellets (dark red), salp fecal pellets (dark blue), krill stomachs (light red), salp stomachs (light blue), and plankton (green). The plot on the right hand side shows the ASVs which were found to be of importance and which are color-coded corresponding to the taxa they were assigned to on the taxonomic level of 'Class'.

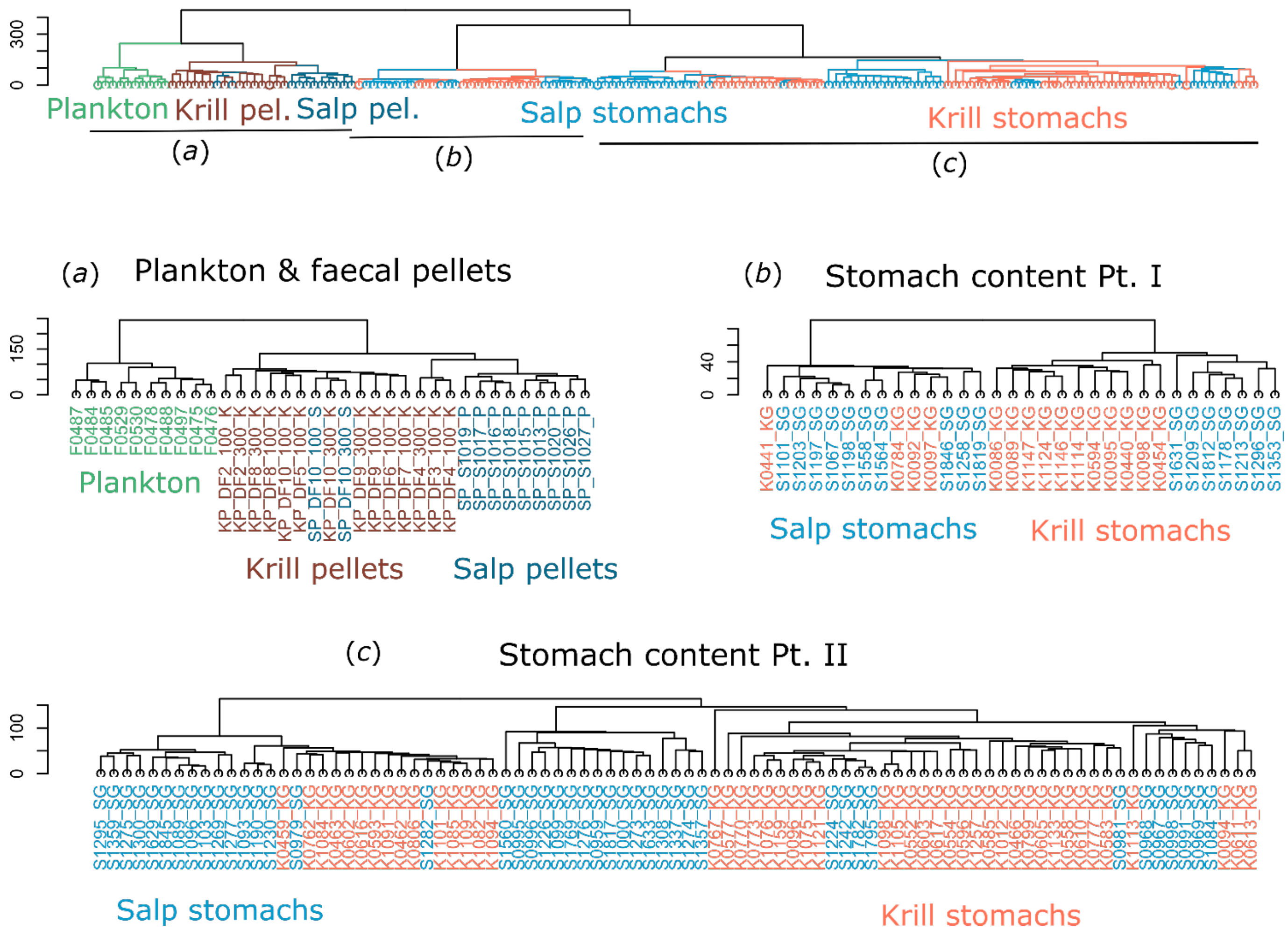

Supplementary Figure 3: Dendrogram of the unsupervised clustering results using Euclidean distance. The top dendrogram shows the clustering results of all samples with plankton in green, krill fecal pellets in dark red, salp fecal pellets in dark blue, salp stomach content in light blue and krill stomach content in light red (from left to right). The following dendrograms zoom into the main branches of the upper panel. From left to right and top to bottom: (a) the first main branch to the left including plankton and fecal pellet samples, (b) the left part of the second main branch including both salp and krill stomach samples, and (c) the right part of the second main branch at the bottom, also containing salp and krill stomach samples.

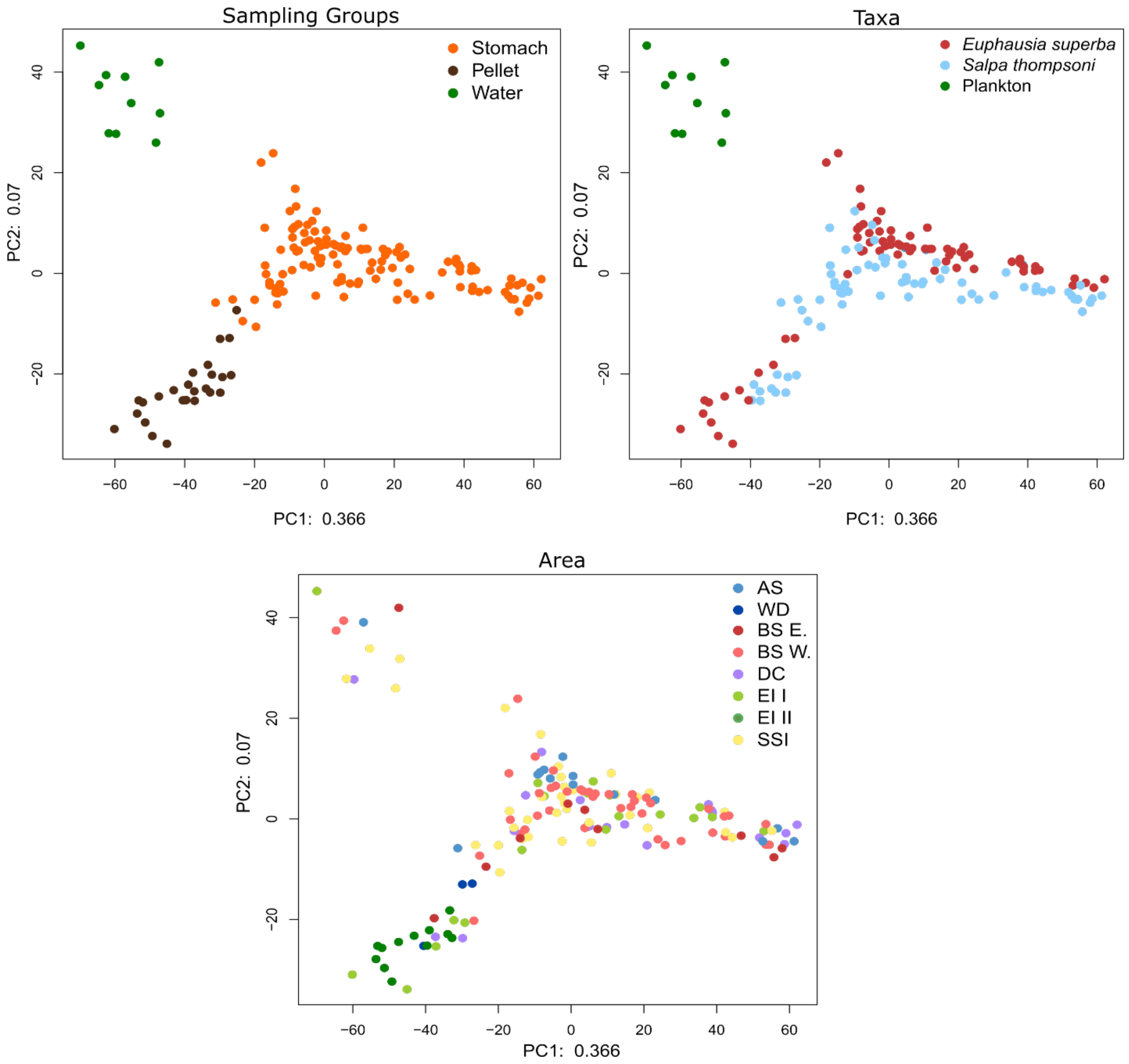

Supplementary Figure 4: Principal component analysis (PCA) of related metadata. Results of the PCA of the refined and centered-log-ratio transformed sequencing dataset including 156 samples and 1765 ASVs. The metadata variables sampling group (water column, stomach content, fecal pellets), taxa (plankton, krill, salps), and area were mapped on the PCA biplot to identify potential clusters. Area abbreviations refer to Supplementary. Table 2: AS = Antarctic Sound, WD = Weddell Sea, BS E. = Bransfield Strait East, BS W. = Bransfield Strait West, DC = Deception Island, EI I = Elephant Island I, EI II = Elephant Island II, SSI = South Shetland Islands.

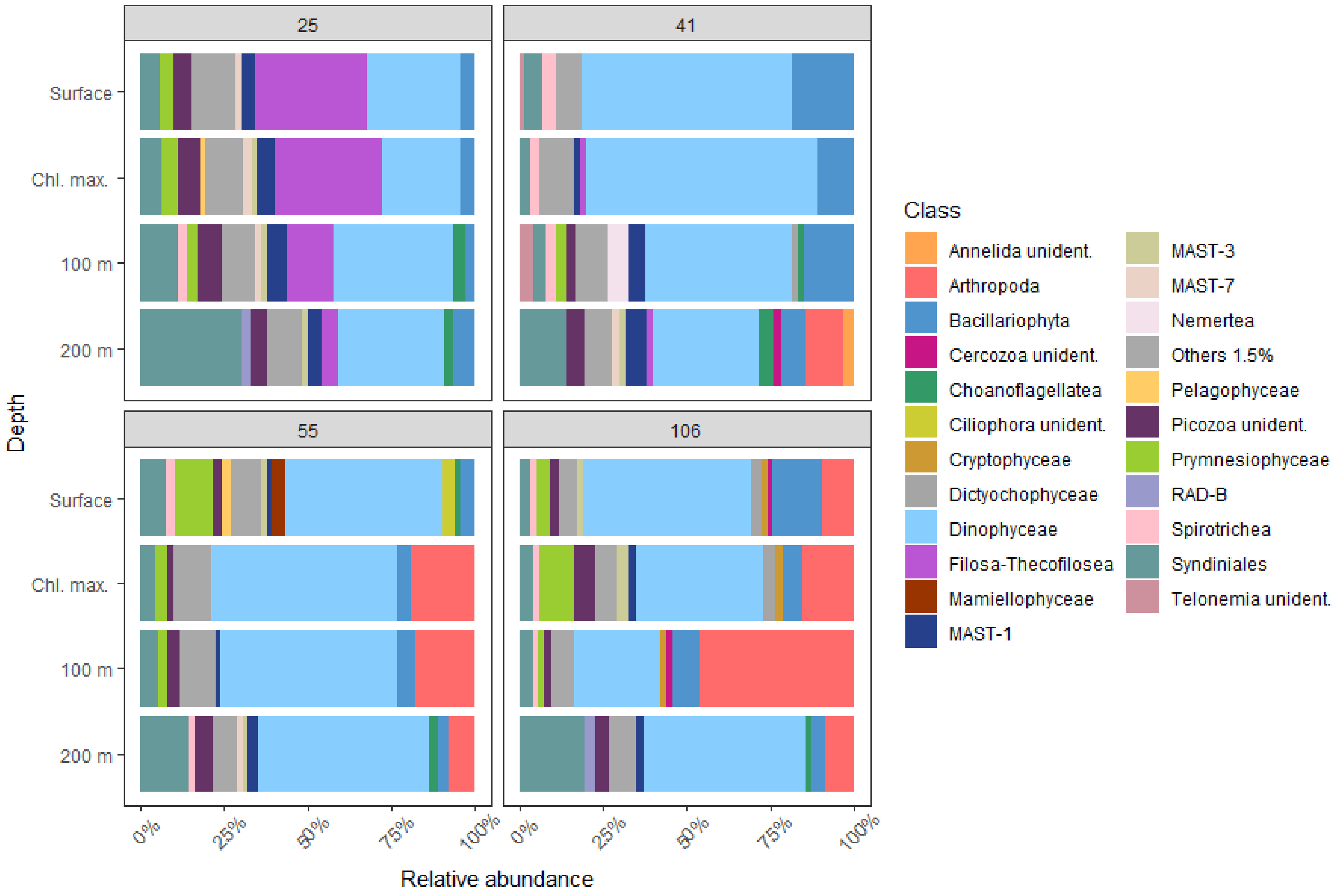

Supplementary Figure 5: Relative abundance of the 18s libraries of the plankton community on the taxonomic level of ‘Class’ across four different depths at four of the ten sampled stations. Water samples were collected at the surface (3–14 m), in the chlorophyll maximum layer (Chl. max., 25–60 m), and at 100 and 200 m, respectively.

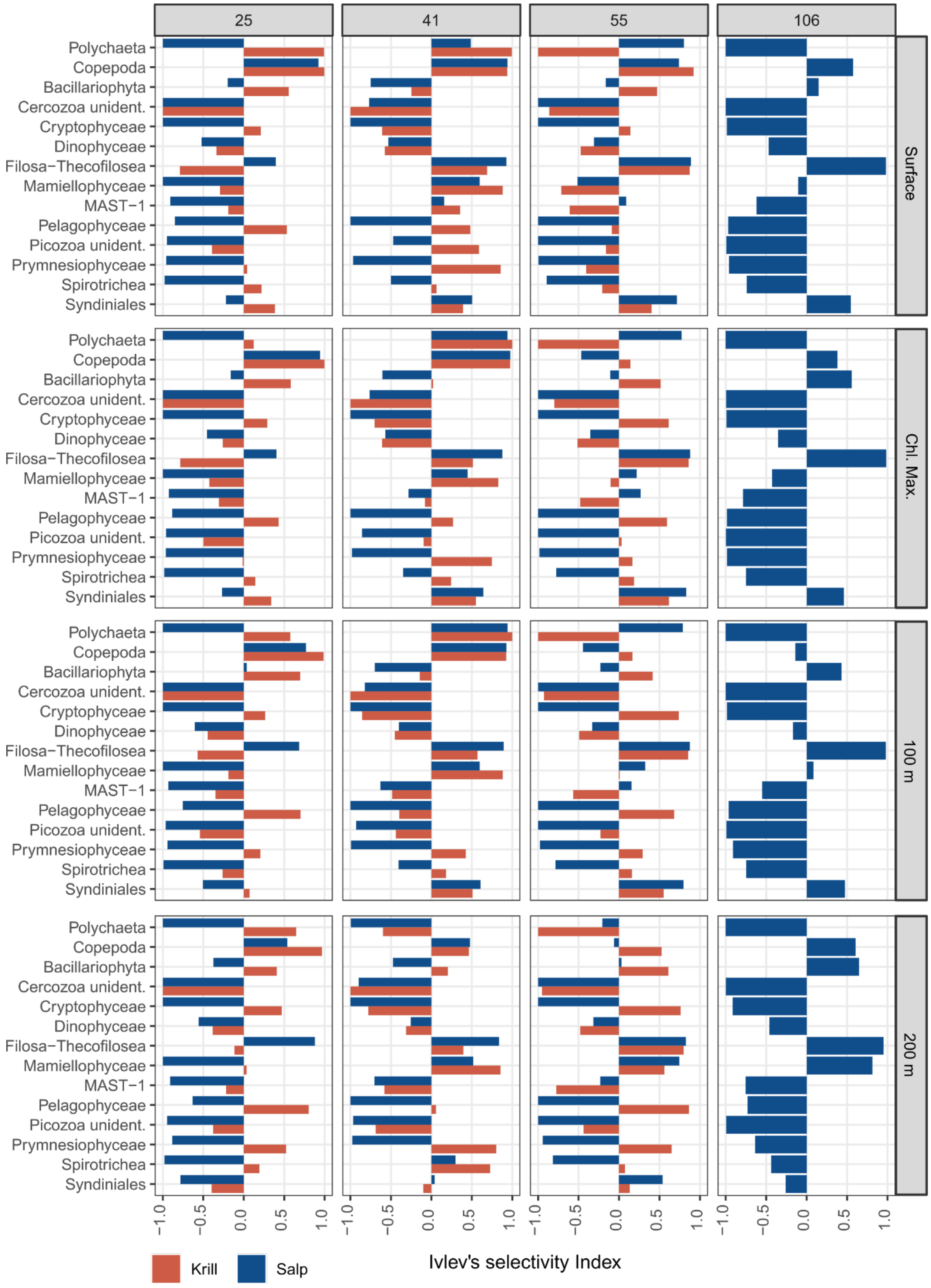

Supplementary Figure 6: Feeding selectivity of krill and salps across different depths. Ivelv's selectivity index indicating avoidance (–1) and preference (+1) of krill and salps in relation to the relative abundance of the plankton community across four depths layers (surface, chlorophyll maximum, 100 m and 200 m) at four of the ten sampling stations (25, 41, 55, 106). The selectivity index for krill is shown in red, the selectivity of salps is depicted in blue. At station 106 only salps were caught.

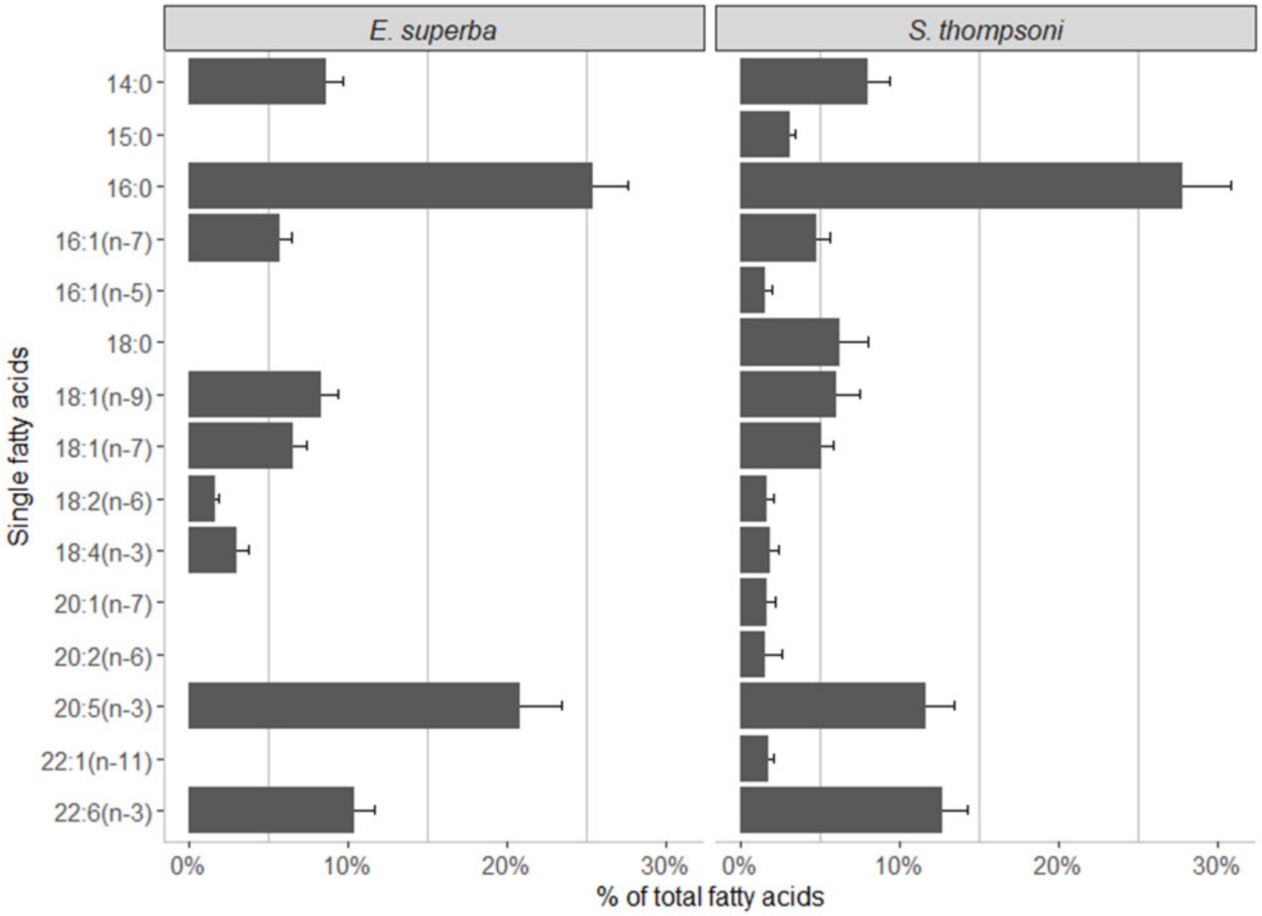

Supplementary Figure 7: Fatty acids identified from the tissue of krill (*E. superba*) and salps (*S. thompsoni*). Only fatty acids with a relative abundance of more than 1.5% in the respective samples of krill (left, n = 21), and salps (right, n = 22) are shown. The share of single fatty acids is given as percentage of the total fatty acids. Error bars represent standard errors.

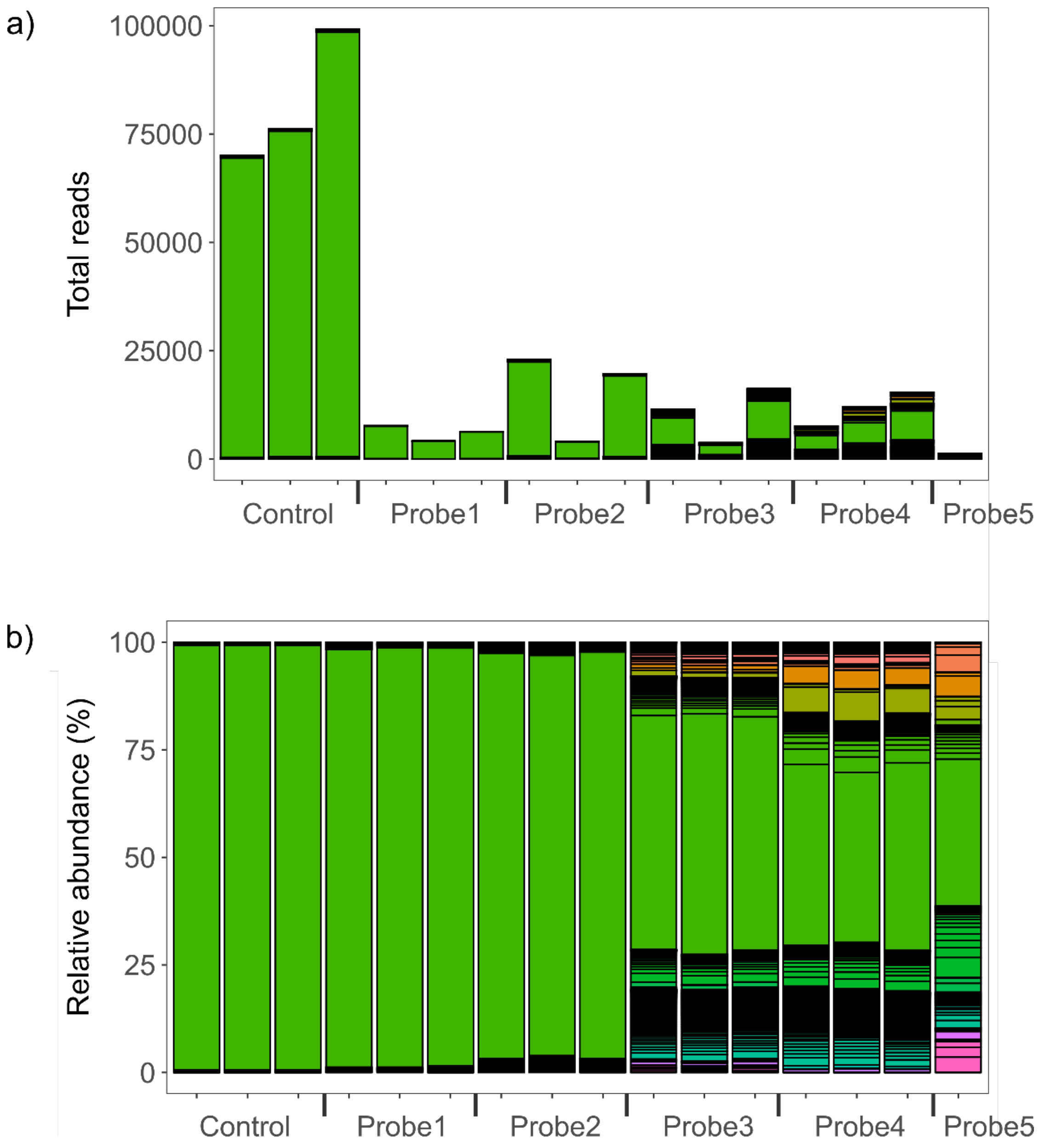

Supplementary Figure 8: Results of the initial blocking probe tests. The bar plot shows the sequencing results for the five tested probes and a control without probe, analyzed in triplicates on the x-axis. The number of total reads per samples (a) and relative abundance of sequences per samples (b) are shown on the y-axis. Predator DNA (Antarctic krill, or closely related species of the order Eucarida) are depicted in green.

Supplementary References

1 Ludwig, W. et al. ARB: a software environment for sequence data. *Nucleic Acids Res.* **32,** 1363-1371 (2004).
