# Supplementary material for: Selective feeding in Southern Ocean key grazers—diet composition of krill and salps
Source: Commun Biol. 2021 Sep 10;4:1061. doi: 10.1038/s42003-021-02581-5 (PMC8433442; doi:10.1038/s42003-021-02581-5)
Supplement: Supplementary file 3 — Description of Additional Supplementary Files [file 42003_2021_2581_MOESM3_ESM.pdf]

Description of Additional Supplementary Files

File name: Supplementary Movie 1.

Description: Salp performing back-flush.

File name: Supplementary Movie 2.

Description: Krill holding onto a salp chain.
