# Supplementary material for: Selective feeding in Southern Ocean key grazers—diet composition of krill and salps
Source: Commun Biol. 2021 Sep 10;4:1061. doi: 10.1038/s42003-021-02581-5 (PMC8433442; doi:10.1038/s42003-021-02581-5)
Supplement: Supplementary file 6 — Reporting Summary [file 42003_2021_2581_MOESM6_ESM.pdf]

nature research

Corresponding author(s): Nora-Charlotte Pauli
Morten H. Iversen
Bettina Meyer

Last updated by author(s): Aug 4, 2021

# Reporting Summary

Nature Research wishes to improve the reproducibility of the work that we publish. This form provides structure for consistency and transparency in reporting. For further information on Nature Research policies, see our Editorial Policies and the Editorial Policy Checklist.

## Statistics

For all statistical analyses, confirm that the following items are present in the figure legend, table legend, main text, or Methods section.

| n/a | Confirmed | |
|---|---|---|
| ☐ | ☒ | The exact sample size ($n$) for each experimental group/condition, given as a discrete number and unit of measurement |
| ☐ | ☒ | A statement on whether measurements were taken from distinct samples or whether the same sample was measured repeatedly |
| ☐ | ☒ | The statistical test(s) used AND whether they are one- or two-sided<br>*Only common tests should be described solely by name; describe more complex techniques in the Methods section.* |
| ☐ | ☒ | A description of all covariates tested |
| ☐ | ☒ | A description of any assumptions or corrections, such as tests of normality and adjustment for multiple comparisons |
| ☐ | ☒ | A full description of the statistical parameters including central tendency (e.g. means) or other basic estimates (e.g. regression coefficient) AND variation (e.g. standard deviation) or associated estimates of uncertainty (e.g. confidence intervals) |
| ☐ | ☒ | For null hypothesis testing, the test statistic (e.g. $F$, $t$, $r$) with confidence intervals, effect sizes, degrees of freedom and $P$ value noted<br>*Give P values as exact values whenever suitable.* |
| ☒ | ☐ | For Bayesian analysis, information on the choice of priors and Markov chain Monte Carlo settings |
| ☒ | ☐ | For hierarchical and complex designs, identification of the appropriate level for tests and full reporting of outcomes |
| ☒ | ☐ | Estimates of effect sizes (e.g. Cohen's $d$, Pearson's $r$), indicating how they were calculated |

*Our web collection on statistics for biologists contains articles on many of the points above.*

## Software and code

Policy information about availability of computer code

| | |
|---|---|
| Data collection | 18S metabarcoding was performed on an Illumina MiSeq platform |
| Data analysis | R, version 3.5.2 & 3.6.1<br>dada2 package in R, version 1.1.1, Callahan et al. 2016<br>DADA2 analysis online tutorial, https://benjjneb.github.io/dada2/tutorial.html<br>Primer trimming using cutadapt, version 1.9, Martin 2011<br>Taxonomy assignment using PR2-Protist Reference Ribosomal Database, version 4.12.0, https://github.com/pr2database<br>Scripts for multivariate analysis from Gloor et al 2016, Bian et al. 2017 and https://github.com/ggloor/CoDaSeq<br>zCompositions R package, version 1.3.4, Palarea-Albaladejo & Fernandez 2015<br>ALDEx2 package in R, version 1.19.4, Fernandes et al 2013<br>propr package in R, version 4.2.6, Quinn et al 2017<br>easyCODA package in R, version 0.31.1, Greenacre 2018 |

For manuscripts utilizing custom algorithms or software that are central to the research but not yet described in published literature, software must be made available to editors and reviewers. We strongly encourage code deposition in a community repository (e.g. GitHub). See the Nature Research guidelines for submitting code & software for further information.

## Data

Policy information about availability of data

All manuscripts must include a data availability statement. This statement should provide the following information, where applicable:

- Accession codes, unique identifiers, or web links for publicly available datasets
- A list of figures that have associated raw data
- A description of any restrictions on data availability

Data are available in the supplementary material of this manuscript. The raw, primer trimmed paired-end sequencing reads were deposited in the European Nucleotide Archive (ENA) and will be accessible under the project number PRJEB40056. The two movies are uploaded as part of the supplementary material and are available at fisgshare doi 10.6084/m9.figshare.14216378

# Field-specific reporting

Please select the one below that is the best fit for your research. If you are not sure, read the appropriate sections before making your selection.

☐ Life sciences ☐ Behavioural & social sciences ☒ Ecological, evolutionary & environmental sciences

For a reference copy of the document with all sections, see nature.com/documents/nr-reporting-summary-flat.pdf

# Ecological, evolutionary & environmental sciences study design

All studies must disclose on these points even when the disclosure is negative.

| | |
|---|---|
| Study description | To assess the implications of a dominance shift from krill to salps on the food web and bioegeochemaical cycles at the western Antarctic Peninsula, we compared the diet composition of Antarctic krill and salps using 18S metabarcoding. Genomic DNA was extracted from water samples, stomach contents of krill and salps, and their faceal pellets. A 436bp long fragment of variable region V4 of the 18S rRNA was amplified and subsequently sequenced on an Illumina MiSeq platform. In addition, fatty acids from tissue samples of krill and salps were studied as long-term dietary markers. |
| Research sample | The majority of biomass of Antarctic krill is located in the SW sector of the Atlantic Ocean with particularly high densities around the western Antarctic Peninsula (WAP). Also salps have shown an increasing abundance in this region over the past decades. We sampled at 26 different stations in six main regions along WAP to obtain a representative sampling group of both species. Krill samples included an randomly distributed amount of male, female and juvenile individulas with a size range from 28-65 mm, mean = 46.86 mm. Salp were represeetd by a randomly chosen group of solitary and aggregate stages from 1 to 100mm in size, mean = 26 mm. |
| Sampling strategy | Oblique net hauls were conducted using Isaak-Kidd Midwater or Rectangular Midwater trawls in the upper 200m of the water column to collect krill and salps. The samples size of ten individuals per species and station, resuting in a total of 61 krill stomach and 60 salp stomach samples, was chosen based on sampling and transport possibilities and available laboratory and sequencing capacities under consideration of the statistical power.<br>A CTD rosette was used to collect water samples from the depth of the chlorophyll maximum to get a random sample of the ambient plankton community. In addition, at four stations water samples were collected at four depths (surface, chl. max., 100 and 200 m). |
| Data collection | Genomic DNA was extracted, amplified and sequenced on an Illumna MiSeq platform (NCP, KM). Raw sequences were evaluated and processed based on available scripts (NCP, SN, KM).<br>Fatty acids were extracted from tissue samples based on an exsting protocol by Kattner & Fricke and processed on an Agilent 6890N gas chromatograph (NCP, MG). Data analysis was performed using a set of different software tools (see above, NCP, SN, KM, MHI, BM). |
| Timing and spatial scale | All samples were collected during the research cruise PS112 with RV Polarstern from March to May 2018 along the western Antarctic Peninsula in the Southern Ocean between 60° 44′53 S to 63°59′16 S and 53°55′39 W to 60°31′57 W. |
| Data exclusions | Raw sequencing reads with length of more than 450bp or less than 320bp were removed as they were not in the range of the targeted insert. In addition, reads with less than 300 read counts overall and amplicon sequence variants with less than 100 read counts were removed. |
| Reproducibility | Sequencing of krill stomach content samples was performed in technical triplicates per biological sample and replicates yielded comparable results. All necessary protocols and laboratory procedures to reproduce 18S metabarcoding are mentioned in the manuscript, along with references to all applied software and codes. |
| Randomization | Individuals of krill and salps for stomach content and fatty acid analyses were randomly chosen from the available catch. No further randomization procedures were necessary during this study. |
| Blinding | Blinding was not relevant in this study. |

Did the study involve field work? ☒ Yes ☐ No

## Field work, collection and transport

| | |
|---|---|
| Field conditions | Ship-based sampling was generally conducted during calm weather conditions. Mean wind forces were 5-6 Bft with mainly westerly winds and sea states of 1-2 m. Details on the field conditions during PS112 can be accessed at https://doi.org/10.2312/BzPM_0722_2018. |
| Location | Field work was conducted along the western Antarctic Peninsula in the Southern Ocean. Longitude and latitude data for all stations are presented in Figure 1 and Supplementary Table 1 of the manuscript. |
| Access & import/export | All field work was conducted in compliance with national and international regulations and necessary authorization was granted by the german enviromental agency. |
| Disturbance | No particular disturbance was caused during sampling. |

# Reporting for specific materials, systems and methods

We require information from authors about some types of materials, experimental systems and methods used in many studies. Here, indicate whether each material, system or method listed is relevant to your study. If you are not sure if a list item applies to your research, read the appropriate section before selecting a response.

### Materials & experimental systems

| n/a | Involved in the study |
|---|---|
| ☒ | ☐ Antibodies |
| ☒ | ☐ Eukaryotic cell lines |
| ☒ | ☐ Palaeontology and archaeology |
| ☐ | ☒ Animals and other organisms |
| ☒ | ☐ Human research participants |
| ☒ | ☐ Clinical data |
| ☒ | ☐ Dual use research of concern |

### Methods

| n/a | Involved in the study |
|---|---|
| ☒ | ☐ ChIP-seq |
| ☒ | ☐ Flow cytometry |
| ☒ | ☐ MRI-based neuroimaging |

## Animals and other organisms

Policy information about studies involving animals; ARRIVE guidelines recommended for reporting animal research

| | |
|---|---|
| Laboratory animals | No laboratory animals were used in this study. |
| Wild animals | Antarctic krill (Euphausia super), Salps (Salpa thompsoni) |
| Field-collected samples | Wild caught Antarctic krill and salps were measured, sexed and staged within five minuted after catch and subsequently frozen in liquid nitrogen. Salps for facal pellet production experiments were kept for a maximum of 12 hours in ambient seawater at ambient temperature (1°C), subsequently sexed, staged and measured before being frozen in liquid nitrogen. |
| Ethics oversight | No ethical approval was neccessary. |

Note that full information on the approval of the study protocol must also be provided in the manuscript.
